# Alzheimer’s Disease Polygenic Risk in the LEADS Cohort

**DOI:** 10.1002/alz.092118

**Published:** 2025-01-03

**Authors:** Kelly N. Nudelman, Julian V. Pentchev, Trever Jackson, Ani Eloyan, Jeffrey L. Dage, Tatiana M. Foroud, Dustin B. Hammers, Maria C. Carrillo, Bradford C. Dickerson, Gil D. Rabinovici, Liana G. Apostolova, the LEADS Consortium

**Affiliations:** ^1^ Department of Medical and Molecular Genetics, Indiana University School of Medicine, Indianapolis, IN USA; ^2^ Indiana Alzheimer’s Disease Research Center, Indianapolis, IN USA; ^3^ Indiana University, Bloomington, IN USA; ^4^ Department of Biostatistics, Brown University, Providence, RI USA; ^5^ Department of Neurology, Indiana School of Medicine, Indianapolis, IN USA; ^6^ Indiana University School of Medicine, Indianapolis, IN USA; ^7^ Alzheimer’s Association, Chicago, IL USA; ^8^ Massachusetts General Hospital and Harvard Medical School, Boston, MA USA; ^9^ Massachusetts Alzheimer’s Disease Research Center, Massachusetts General Hospital, Boston, MA USA; ^10^ Department of Neurology, Harvard Medical School, Boston, MA USA; ^11^ UCSF Alzheimer’s Disease Research Center, San Francisco, CA USA; ^12^ Department of Neurology, Memory and Aging Center, University of California San Francisco, San Francisco, CA USA; ^13^ Department of Radiology and Imaging Sciences, Indiana University School of Medicine, Indianapolis, IN USA

## Abstract

**Background:**

Currently, it is unclear to what extent late‐onset Alzheimer’s disease (AD) risk variants contribute to early‐onset AD (EOAD). One method to clarify the contribution of late‐onset AD genetic risk to EOAD is to investigate the association of AD polygenic risk scores (PRS) with EOAD. We hypothesize that in the Longitudinal Early‐Onset Alzheimer’s Disease Study (LEADS), EOAD participants will have greater PRS than early‐onset amyloid‐negative cognitively‐impaired participants (EOnonAD) and controls, and investigate the association of AD PRS with age of disease onset (AoO) and cognitive performance.

**Methods:**

GWAS data was generated for LEADS participants, including those with EOAD, EOnonAD, and controls, with the Illumina Global Screening Array. A PRS was calculated using the 31 SNPs and weights published previously by Desikan et al. (2017) for LEADS participants with imputed GWAS data (N = 369). Logistic regression models including age, sex, PRS, and genetic ancestry principal components were tested to identify predictors of EOAD (N = 210) vs. EOnonAD (N = 69) and controls (N = 89). ANCOVA models were used to assess group differences in PRS scores. Kaplan‐Meier regression was used to assess differences in EOAD AoO for tertile‐binned PRS groups. Within EOAD, pre‐calculated cognitive domain scores for speed and attention, working memory, episodic memory, language, and visuospatial performance were assessed for correlation with PRS.

**Results:**

The AD PRS was a predictor of EOAD (p = 0.014), with the model explaining 10.5% of variance (X2 = 40.971, p<0.001). EOAD participants had higher PRS scores (mean = 0.0012, standard deviation (SD) = 0.015) compared to EOnonAD and controls (mean = ‐0.0018, SD = 0.015) (F = 6.602, p = 0.011). Survival analysis indicated no significant differences in EOAD AoO between PRS groups (X2 = 3.396, p = 0.183). In the EOAD group, PRS was associated with cognitive scores for speed and attention (r = 0.204, p = 0.007), language (r = 0.230, p = 0.002), and visuospatial performance (r = 0.166, p = 0.037).

**Conclusions:**

In the LEADS cohort, AD PRS is a predictor for EOAD, and is associated with cognitive performance, but does not predict EOAD AoO. This suggests that while late onset AD‐associated genetic variants contribute to disease risk and processes, they do not account for a large portion of disease risk, and do not explain differences in disease AoO in the LEADS cohort.

